# Reliability and validity study of the Indonesian Smartphone Application-Based Addiction Scale (SABAS) among college students

**DOI:** 10.1016/j.heliyon.2022.e10403

**Published:** 2022-08-24

**Authors:** Ira Nurmala, Siti Rahayu Nadhiroh, Iqbal Pramukti, Laila Wahyuning Tyas, Afina Puspita Zari, Mark D. Griffiths, Chung-Ying Lin

**Affiliations:** a Department of Epidemiology Population Biostatistics and Health Promotion Behavioral Sciences, Faculty of Public Health, Universitas Airlangga, Indonesia; b Department of Nutrition, Faculty of Public Health, Universitas Airlangga, Indonesia; c Department of Community Health Nursing, Faculty of Nursing, Universitas Padjadjaran, Indonesia; d International Gaming Research Unit, Psychology Department, Nottingham Trent University, Nottingham, UK; e Institute of Allied Health Sciences, College of Medicine, National Cheng Kung University, Tainan 701401, Taiwan; f Department of Occupational Therapy, College of Medicine, National Cheng Kung University, Tainan 701401, Taiwan; g Biostatistics Consulting Center, National Cheng Kung University Hospital, College of Medicine, National Cheng Kung University, Tainan 701401, Taiwan; h Department of Public Health, College of Medicine, National Cheng Kung University, Tainan 701401, Taiwan

**Keywords:** Good health and wellbeing, Smartphone dependence, Smartphone application-based addiction scale (SABAS), Nomophobia, Psychometric testing

## Abstract

**Background/Objective:**

Smartphone addiction, smartphone dependence, and compulsive smartphone use all describe similar phenomena that can cause problems in everyday daily life in many countries worldwide. Most scholars agree that it is the applications on smartphones that individuals have problems with rather than the smartphone itself. For this reason, smartphone application-based addiction is an issue of concern and one instrument has been specifically developed to assess this risk, namely, the Smartphone Application-Based Addiction Scale (SABAS). Although the SABAS has been translated into a number of languages, it has not been translated or validated into Indonesian.

**Methods:**

The SABAS was translated into Bahasa Indonesian utilizing a cross-cultural method to ensure its linguistic validity. The linguistic validity of the Indonesian SABAS was ensured using international standard translation guidelines. Moreover, reliability and validity testing of the translated Indonesian SABAS were carried out using Cronbach’s α, McDonald’s ω, confirmatory factor analysis (CFA), and correlations with psychometric scales assessing psychological distress and nomophobia.

**Results:**

Using a sample of 458 participants (mean age = 22.46 years), reliability tests showed that the Indonesian SABAS was acceptable (Cronbach α = 0.74; McDonald’s ω = 0.79). Construct validity of the Indonesian SABAS was supported by satisfactory CFA fit indices; concurrent validity supported by good correlations with psychological distress (r = 0.50) and nomophobia (r = 0.61).

**Conclusions:**

The Indonesian version of SABAS is valid and reliable to be used for assessing the risk of smartphone application-based addiction in college students.

## 1 Introduction

In daily life, smartphones provide ubiquitous convenience because they now have sophisticated computing and connectivity capabilities (Rashvand and Hsiao, 2015). Every smartphone user has different goals, and various researchers have reported that smartphones have many benefits for both social and health purposes (Ching et al., 2015). However, among a small minority of individuals, excessive smartphone use can lead to psychosocial problems where users appear to become dependent on their smartphones (Arthy et al., 2019). Smartphone addiction, smartphone dependence, and compulsive smartphone use are commonly used terms to describe similar phenomena that can cause problems in individuals daily lives (Kwon and Paek, 2016). Several studies have reported relatively high rates of smartphone addiction such as 14.2% in South Korea (Kwon et al., 2013) and 37.9% in China (Wang et al., 2015). In Europe, these prevalence rates are much higher (12.5%–21.5%) (Lopez-Fernandez, 2017) although it should be noted that most studies comprise relatively small non-representative convenience samples. Young adults appear to be a group at higher risk of smartphone addiction. Young adults can become very attached to their smartphones, and further develop psychological distress (Johnson et al., 2020; Yam et al., 2019).

Concerns relating to problematic screen device use have been reported in numerous studies. For example, a study in South Korea reported that most children use screen devices for more than an hour every day without parental supervision, as well as using screen devices during meal times, resulting in children having decreased attention deficits (Hye et al., 2021). In Indonesia (where the present study was carried out), telecommunications statistics indicate that the percentage of the population using smartphones increased to 63.53% by 2019 (BPS-Statistics Indonesia, 2019). The increase in smartphone use is mirrored by an increase in the population accessing the internet which rose to 47.69% from 21.98% in 2019 (BPS-Statistics Indonesia, 2019). Survey results regarding online use from the Ministry of Communication and Informatics (BPS-Statistics Indonesia, 2019) echoes prior research. For example, Yildirim et al. (2016) reported 42.6% of their Turkish sample (537) had nomophobic behavior because they felt an “irrational” fear when not being able to use their smartphones.

The excessive intensity of smartphone use can have a negative impact among a minority of individuals (Hanafi et al., 2019), especially young adults. Several studies claim that addiction to smartphones has negative consequences or impacts for individuals. According to research conducted by Herrero et al. (2019), smartphone addiction can cause individuals to experience poor psychological adjustments, problems at work, and increased conflicts with family or friends. It can also increase the potential for individuals to be exposed to cyberbullying from social media (Stone, 2020).

Early detection as the first step towards preventing smartphone addiction is necessary (Hanafi et al., 2019). For this reason, a brief and robust instrument is needed to assess whether an individual is at risk of being addicted to smartphones. The instrument used in the present study was the Smartphone Application-Based Addiction Scale (SABAS; Csibi et al., 2018). The SABAS is an instrument that assesses the risk of addiction to applications accessed via smartphone (Csibi et al., 2018). The instrument was developed by Csibi et al. (2018) and comprises six items based on the components model of addiction Griffiths (2005). The SABAS has been validated in various languages including, English (Csibi et al., 2018), Hungarian (Szabo et al., 2017), Italian (Soraci et al., 2021), Persian (Lin et al., 2019), Arabic (Vally and Alowais, 2020), and Chinese (Chen et al., 2020a, Chen et al., 2020b, Chen et al., 2020c; Leung et al., 2020; Yam et al., 2019). All previous translation studies have reported that the SABAS is a valid and reliable scale.

However, there is no previous translation and validation of the SABAS into Bahasa Indonesian. There is much need for a validated version of the SABAS into Bahasa Indonesian due to the increased number of adolescents using smartphones and to monitor the problematic use of smartphone among this population. Based on the 2019 final report by the Ministry of Communications and Information Technology of the Republic of Indonesia, the report stated that internet users in Indonesia have reached more than half of the Indonesia's total population of 268.2 million (i.e., approximately 150 million internet users). It was also reported that there were 130 million social media users mainly accessing social media content via their smartphones (Ministry of Communication and Information, 2019). Other survey data reports that 62.8% of people in Indonesia were using smartphones in 2020 (BPS-Statistics Indonesia, 2020).

Differences in value structures and social customs can dramatically affect how a sample responds to surveys, questionnaires, or other research instruments even with the best translations (Beaton et al., 2000). Translating a psychometric scale from one language to another is not a simple task and should reflect local culture and customs (Kim et al., 2012). This is because each language has characteristics and uniqueness as well as diverse grammatical structures (Yuk et al., 2020). Further translation studies of Smartphone Application-Based Addiction Scale in various countries using cross-cultural methods are much needed, especially in Indonesia where there are 128.03 million teenagers (Jayani, 2021). This number is large enough to justify the need for a translation of the SABAS using a cross-cultural method that still pays attention to linguistic, conceptual, and technical equivalence (Yuk et al., 2020).

It is not psychometrically sound and practical to adapt a scale without considering the cultural and linguistic differences existing between the scale's context of origin and the new context. Moreover, the translation process is a major challenge especially in studies that aim to compare psychometric scales between countries (Cheung et al., 2020). The translation of the SABAS into Indonesian using an Indonesian cultural approach has never been done. For this reason, the present research is a cross-sectional study that translated and validated the SABAS into Bahasa Indonesian that was adapted to the Indonesian cultural context by following a systematic translation method.

## 2 Methods

### 2.1 Participants

The research participants were all active students at Indonesian tertiary institutions studying at various levels, from bachelor's degrees to master's degrees. The participants in the present study were 458 respondents. The inclusion criteria in the present cross-sectional survey study were being an active student status and being willing to participate. Participants were recruited through the university databases from June 29 to December 29, 2021. Study approval was obtained from the Health Research Ethics Commission, Faculty of Nursing, Universitas Airlangga (registration number: 2318-KEPK). For confirmatory factor analysis (CFA), the general rule of thumb is that the sample size of 200 is sufficient (Kline, 2011). Therefore, the sample size in the present study exceeded this greatly.

### 2.2 Study design

The present study was a cross-sectional study that translated and validated the SABAS into Bahasa Indonesian. The scale was adapted to the Indonesian cultural context following a systematic translation method.

### 2.3 Measures

*Smartphone Application Based Addiction Scale (SABAS)*. The SABAS was used as the main instrument to be translated into Bahasa Indonesian. The SABAS assesses the risk of developing smartphone addiction in the past week. The scale comprises six items using a six-point scale to assess each item (1 = strongly disagree, 2 = disagree, 3 = slightly disagree, 4 = slightly agree, 5 = agree, 6 = strongly agree). The total score is calculated by adding up all the scores obtained with a cut-off point of 21 out of 36 which indicates an individual is at risk of smartphone addiction. The higher the score obtained, the higher the level of smartphone addiction risk. The scale has been reported to be reliable with internal consistency of 0.81 for the English version (Csibi et al., 2018), 0.89 for the Italian version (Soraci et al., 2021), 0.86 for the Persian version (Lin et al., 2019), 0.71 for the Arabic version (Vally and Alowais, 2020) and 0.78 for the Chinese version (Leung et al., 2020). The SABAS was proposed to be associated with psychological distress, therefore, in the present study, two other instruments to address psychological state of adolescents were utilized: the 21-item *Depression, Anxiety, and Stress Scale* (DASS-21) and the *Nomophobia Questionnaire* (NMPQ). Moreover, both DASS-21 and NMPQ have been translated into Indonesian versions with robust psychometric properties (Onie et al., 2020; Rangka et al., 2018). Therefore, the DASS-21 and NMPQ used in the present study were Indonesian versions.

*Nomophobia Questionnaire (NMPQ).* The NMPQ assesses the risk of developing nomophobia (i.e., a type of phobia that is afraid of having no smartphone on hand) in the past week. The scale comprises 20 items using a seven-point scale to assess each item (1 = strongly disagree, 7 = strongly agree). The total score is calculated by adding up all the scores obtained with a cut-off point of 60 out of 140 which indicates an individual has moderate level of nomophobia. The scale has been reported to be reliable with internal consistency of 0.94–0.95 for the English version (Lee et al., 2018; Yildirim and Correia, 2015), 0.92 for the Persian version (Lin et al., 2018), 0.96 for the European Portuguese version (Galhardo et al., 2020), and 0.93 for the Indonesia version (Rangka et al., 2018).

*Depression, Anxiety, Stress Scale (DASS-21)*. The DASS-21 assesses the risk of developing psychological distress (i.e., depression, anxiety, and stress) in the past week. The scale comprises 21 items using a four-point scale to assess each item (0 = did not apply to me at all, 3 = applied to me very much or almost all the time). The total score is calculated by adding up all the scores obtained and multiplying by 2 with a cut-off point of 59 out of 126 which indicates an individual has moderate level of psychological distress. The scale has been reported to be reliable with internal consistency of 0.78–0.89 for the English version (Coker et al., 2018), 0.800.92 for the Chinese version (Wang et al., 2016), 0.76–0.91 for the Vietnamese version (Le et al., 2020), and 0.79–0.91 for the Indonesia version (Onie et al., 2020).

### 2.4 Procedure

The present study followed the translation procedures from Cheung et al. (2020) that draws from existing guidelines and recommendation in health and medicine cross-cultural research.

#### 2.4.1 Step 1: Recruit translation team

The first step was to recruit a team of translators. A team of experienced and professionally qualified translators is required. To produce a quality translation, a translation team must consist of a minimum of four translators arranged in pairs with equal skills. In the present study, the researchers recruited four translators divided into mixed-pairs based on expertise and background. The translation team (A) consisted of A1 translators, namely translators who understood issues related to smartphone addiction from Universitas Airlangga and translators A2 who were experts in English translation in the city of Surabaya. The translation team B comprised translators B1 who understood the related issues from Universitas Padjadjaran and translators B2 were English translators from Bandung.

#### 2.4.2 Step 2: Forward translation

Only the partner from the first translation team (team A) was involved. Each translator from the translation team A, namely translator A1 (who understood the issue) and translator A2 (English expert). The two translators carried out the translation of the SABAS in the original English version, namely the original document (Document 0), into the Bahasa version. For one week, the two translators submitted documents resulting from the translation into Bahasa Indonesian, namely Document 1 which was produced by translator A1 and Document 2 which was produced by translator A2. After that, a committee approach was taken to evaluate the differences and an agreement was reached to produce a combined document (Document 3). Detailed information of the forward translation is provided in Table 1.

**Table 1:** Process of *Forward Translation* into Bahasa by translation team A

Original Document in English		*Forward translation* into Bahasa Indonesia		
		Translator A1	Translator A2	Translator A1
1.	My smartphone is the most important thing in my life	Ponsel saya adalah hal terpenting dalam hidup saya	*Smartphone* adalah hal terpenting dalam hidup saya	*Smartphone* adalah hal terpenting dalam hidup saya
2.	My smartphone use results in conflicts	Penggunaan ponsel saya menghasilkan sebuah konflik	*Saya menggunakan smartphone* terlalu sering sehingga menyebabkan konflik	Aktivitas saya menggunakan *smartphone* menyebabkan konflik
3.	Preoccupying myself with my smartphone is a way of changing my mood	Menyibukkan diri dengan ponsel saya adalah cara mengubah suasana hati saya	Menyibukkan diri dengan *smartphone* dapat mengubah suasana hati saya	Menyibukkan diri dengan *smartphone* adalah cara untuk mengubah suasana hati saya
4.	Over time, I fiddle around more and more with my smartphone	*Saya semakin* sering bermain ponsel secara berlebihan	*Saya menyia*-nyiakan waktu hanya untuk bermain *smartphone*	*Saya menghabiskan* waktu terus-menerus hanya untuk bermain *smartphone*
5.	If I cannot use my smartphone when I feel like, I feel sad	*Jika saya* tidak dapat menggunakan ponsel saya, saya merasa sedih	Ketika tidak menggunakan smartphone saat ingin, saya merasa sedih	Ketika saya tidak dapat menggunakan *smartphone* saat ingin, saya merasa sedih
6.	If I try to cut the time I use my smartphone, I end up using it as much or more than before	*Jika saya* mencoba untuk membatasi waktu saya dengan menggunakan ponsel, saya akhirnya menggunakannya lebih banyak dari sebelumnya	Ketika saya mencoba berhenti menggunakan *smartphone*, saya malah menggunakannya lebih lama atau lebih sering dari sebelumnya	*Jika saya* mencoba mengurangi waktu menggunakan *smartphone*, saya semakin menggunakannya lebih lama atau lebih sering dari sebelumnya

#### 2.4.3 Step 3: Back-translation

The third stage was back-translation, where the second translation team (translation team B) performed a back-translation of the combined translated documents into English independently. At this stage, the translation team B consisting of translator B1 and translator B2, retranslated the combined translation document (Document 3) into English to produce Document 4 (generated by translator B1) and Document 5 (generated by translator B2). The two translators were not given access to the original scale at this stage. Next, a comparison was made between the two to identify differences and the evaluation to be carried out. There was a difference in the back-translation process in the present study, namely in question Item number 4 on the SABAS. Detailed information of the forward translation is provided in Table 2.

**Table 2:** Process of *Back-Translation* into English version by Translation Team B.

No.	*Back-translation* into Bahasa Inggris		*Final translation* in Bahasa Indonesia
	Translator B1	Translator B2	
1.	My smartphone is the most important thing in my life	My smartphone is the most important thing in my life	*Smartphone* adalah hal terpenting dalam hidup saya
2.	My activity using a smartphone leads to conflict	Using smartphones in my activity causes conflict	Aktivitas saya menggunakan *smartphone* menyebabkan konflik
3.	Occupying myself with a smartphone is a way to change my mood	Keeping myself busy with my smartphone is a way to change my mood	Menyibukkan diri dengan *smartphone* adalah cara untuk mengubah suasana hati saya
4.	I spent a lot of time just to play with the smartphone	I spend time constantly just playing with my smartphone	*Saya menghabiskan* waktu terus-menerus hanya untuk bermain *smartphone*
5.	When I could not use the smartphone when I want to, I felt sad	When I can't use my smartphone when I want to, I feel sad	Ketika saya tidak dapat menggunakan *smartphone* saat ingin, saya merasa sedih
6.	If I tried to reduce the time I use the smartphone, I ended up using it longer or more often than before	If I try to reduce the time I use my smartphone, I end up using it longer or more often than before	*Jika saya* mencoba mengurangi waktu menggunakan *smartphone*, saya semakin menggunakannya lebih lama atau lebih sering dari sebelumnya

#### 2.4.4 Step 4: Committee consolidation

At this stage, a committee consisting of researchers and the entire translation team came together to examine similarities and differences between instrument sources and back-translation documents. The documents examined were Document 0 (English version), Documents 1, 2, and 3 (forward translation) and Documents 4 and 5 (back-translation). To overcome the differences in the back-translation stage, the researchers and all translators agreed to accept the translation and retained it according to the original concept of the scale. As a result, a consolidated document was produced which was used for the psychometric testing.

#### 2.4.5 Step 5: Pilot test and confirming Indonesian SABAS

Trials were carried out to correct errors in the scale and ensure that the final translation results had maintained equivalence before the researchers deployed the instrument in the field. In the present study, trials were conducted with 33 students who were willing to be participants from selected universities in Indonesia on July 14, 2021, and which were distributed online using *Google Forms*. After distributing the scale online, the validity and reliability of the translated SABAS was tested using SPSS software. The internal consistency of the Indonesian SABAS was acceptable (Cronbach's α = 0.753), which means the scale is reliable. The scale was then sent out for formal psychometric testing.

### 2.5 Data analysis for formal psychometric testing

Two types of internal consistency tests were applied; Cronbach’s α and McDonald’s ω. The recommended cutoff indicating good internal consistency for both Cronbach’s α and McDonald’s ω is 0.7 (Nunnally, 1978). Construct validity of the SABAS was then examined using the CFA given that the SABAS has been found to be a unidimensional instrument (Chen et al., 2020; Leung et al., 2020; Lin et al., 2017; Poon et al., 2021; Wu et al., 2017; Yam et al., 2019). In the CFA, a diagonally weighted least squares estimator was used to take care of the ordinal scales used in the SABAS. Fit indices derived from the CFA were then used to evaluate the construct validity of the SABAS. More specifically, the unidimensionality of the SABAS can be supported if the following conditions are satisfied: a nonsignificant χ^2^ test, comparative fit index (CFI) and Tucker-Lewis index (TLI) > 0.9, root mean square error of approximation (RMSEA) and standardized root mean square residual (SRMR) < 0.08 (Lin et al., 2020; Nejati et al., 2021).

Concurrent validity of the SABAS was then examined using structural equation modeling (SEM). Similar to the CFA, the SEM adopted a diagonally weighted least squares estimator. More specifically, the SABAS was proposed to be regressed on both psychological distress (assessed using the DASS-21) and nomophobia (assessed using NMPQ). Both DASS-21 and NMPQ are psychological stress-related instruments. Moreover, based on the Interaction of Person-Affect-Cognition-Execution (I-PACE) model (Brand et al., 2016; Chang et al., 2020; Chen et al., 2021a, Chen et al., 2021b; Chen et al., 2021; Chen et al., 2020), psychological distress was hypothesized to be associated with problematic smartphone use. Therefore, the SABAS score was hypothesized to be positively associated with both DASS-21 and NMPQ scores. The CFA and SEM were conducted using *lavaan* package (Rosseel, 2012).

## 3 Results

### 3.1 Sample characteristics and psychometric properties

The sample for the formal psychometric testing of the Indonesian SABAS had a mean age of 22.46 years (SD = 8.07) with the majority of participants being female (n = 339; 74.0%). Moreover, most of the participants were undergraduates (n = 376; 82.1%). Additional characteristics of the participants are reported in Table 3. Table 4 reports the item and total scores of the SABAS. The item scores of the SABAS were normally distributed (skewness ranged between -0.80 and 0.59; kurtosis ranged between -0.90 and 0.18). Both internal consistency values were acceptable for the SABAS (Cronbach’s α = 0.74 and McDonald’s ω = 0.79).

**Table 3:** Characteristics of the participants (N = 458).

	n (%) or M (SD)
Age in year	22.46 (8.07)
Gender	
*Male*	119 (26.0)
*Female*	339 (74.0)
Study major	
*Health-related*	251 (54.8)
*Non-health-related*	207 (45.2)
Study program	
*Undergraduate*	376 (82.1)
*Postgraduate*	82 (17.9)
Marital status	
*Single*	387 (84.6)
*Married*	68 (14.8)
*Other*	3 (0.6)

**Table 4:** Score distributions and internal consistency for the Smartphone Application Based Addiction Scale (SABAS).

	Mean (SD)	Skewness	Kurtosis	α^a^	ω^b^
SABAS	19.72 (4.96)	0.08	-0.10	0.74	0.79
Item S1	4.28 (1.20)	-0.80	0.18		
Item S2	2.85 (1.15)	0.56	-0.36		
Item S3	3.94 (1.32)	-0.57	-0.43		
Item S4	2.93 (1.38)	0.35	-0.90		
Item S5	2.95 (1.25)	0.29	-0.81		
Item S6	2.78 (1.16)	0.59	-0.37		

a Cronbach’s α.

b McDonald’s ω

### 3.2 Fit indices and SEM analyses

**Figure 1:**
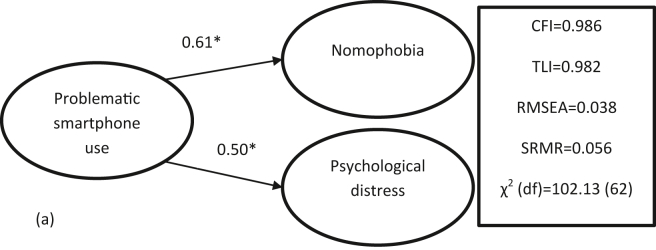
Concurrent validity of the Smartphone Application Based Addiction Scale (assessing problematic smartphone use) with nomophobia and psychological distress via structural equation modeling. CFI = comparative fit index; TLI = Tucker-Lewis index; RMSEA = root mean square error of approximation; SRMR = standardized root mean square residual ∗*p* < .001.

The unidimensionality of the SABAS was fully supported by the fit indices of CFA (*p*-values of χ^2^ = 0.17; CFI = 0.994; TLI = 0.990; RMSEA = 0.031; and SRMR = 0.041). Moreover, the factor loadings for the SABAS were good (ranging between 0.24 and 0.66 for SABAS) (Table 5). The concurrent validity of the SABAS was supported as Figure 1 shows the significant correlations between SABAS and the other two external criterion instruments (i.e., psychological distress assessed using DASS-21 and nomophobia assessed using NMPQ). More specifically, the SEM results showed that problematic smartphone use (assessed using SABAS) was associated with psychological distress (*r* = .50; *p* < .001) and nomophobia (*r* = .61; *p* < .001).

**Table 5:** Psychometric results derived from confirmatory factor analysis on the Smartphone Application Based Addiction Scale (SABAS).

	SABAS
Factor loading	
Item 1	0.48
Item 2	0.42
Item 3	0.63
Item 4	0.66
Item 5	0.60
Item 6	0.64
Fit indices	
χ^2^ (df)	12.89 (9)
p-value	0.17
CFI	0.994
TLI	0.990
RMSEA	0.031
95% CI of RMSEA	0.000, 0.065
SRMR	0.041

CFI = comparative fit index; TLI = Tucker-Lewis index; RMSEA = root mean square error of approximation; SRMR = standardized root mean square residual.

## 4 Discussion

Researchers who do not have a psychometric instrument to assess the risk of smartphone addiction applications that are appropriate to their language and culture can consider two options. These are namely creating new instruments or modifying instruments made in other languages by adapting cross-cultural methods. In the present study, the choice of instrument modification from another language was carried out through the translation process and validation of the SABAS into the Bahasa Indonesian version using a cross-cultural method approach. The present study is the first conducted in Indonesia related to the translation and linguistic validation of the SABAS. A systematic translation following Cheung et al.'s (2020) method was applied during the translation process from English to Bahasa and adapted to Indonesian culture. This process required translators who were competent in smartphone addiction issues and native languages as well as English language experts.

The linguistic validity of the Indonesian SABAS was confirmed with the use of standardized cross-cultural approach (Beaton et al., 2000). Moreover, the pilot testing showed that the linguistically validated Bahasa Indonesian version of the SABAS had good internal consistency. More specifically, Cronbach's α was = 0.753 in the pilot testing and 0.74 in the formal psychometric testing. Although the value was slightly lower than the original version (Szabo et al., 2017), it did not affect the reliability adequacy of the scale. More specifically, the result is consistent with several previous studies (Leung et al., 2020; Vally and Alowais, 2020), which also had a slightly lower internal consistency value than the original version. A potential reason is that the translation may not perfectly replicate the original meanings even though a standardized translation procedure was applied to ensure the linguistic validity of scale items. Consequently, it may be that a translated scale has a lower internal consistency than its original version as has been found in other validation studies in other languages.

Apart from the good internal consistency, the present study showed that the SABAS has a unidimensional structure which concurs with prior studies. More specifically, different language versions of the SABAS (such as the original Hungarian version, English version, and Chinese version) all demonstrated that the SABAS has a unidimensional structure (Chen et al., 2020; Csibi et al., 2016, 2018; Lin et al., 2019; Ndağ and Ünal, 2019; Soraci et al., 2021; Yam et al., 2019). Therefore, the SABAS appears to be assessing a single latent construct (i.e., addiction to smartphone applications) that has been demonstrated across a number of different country populations and particularly among university students.

Given that the unidimensional structure of the SABAS was confirmed among an Indonesian population, the present study further demonstrated how this psychometric scale assessing problematic smartphone applications was associated with poor psychological health (i.e., depression, anxiety, and stress). Previous research has shown that problem internet-related activities have increased among schoolchildren and university students, and that they are associated with greater psychological distress in both groups (Chen et al., 2021; Chen et al., 2020). Furthermore, problematic smartphone/internet use among individuals with schizophrenia seems to affect longitudinal social functioning through poor sleep and self-stigma concerns (Chang et al., 2022), and was shown to mediate the association between self-stigma and anxiety, and the association between self-stigma and stress (Chang et al., 2020). Therefore, the association between problematic smartphone use and psychological distress in the present study appear to concur with the aforementioned research findings.

In the present study, the psychometric testing of the SABAS demonstrated acceptable internal consistency values (Cronbach’s = 0.74 and McDonald’s = 0.79). This is in line with other studies where each of the six SABAS items significantly correlated with all other items in the scale (*p* < 0.01), supported factorability with the internal reliability of the scale checked using Cronbach’s alpha, and demonstrated good reliability (Cronbach alpha = 0.81) (Csibi et al., 2018). In the present study, SABAS correlated with nomophobia and psychological distress. This is supported because the use of social media applications is beneficial in lowering the symptoms of depression (Elhai et al., 2017). Moreover, other studies have found that social media addiction including gaming addiction is associated with high levels of depressive symptoms (e.g., Bányai et al., 2017; Purwaningsih & Nurmala, 2021).

Problematic smartphone use is also associated with psychological distress (Chen et al., 2020). The association of problematic smartphone use with psychological distress may facilitate the development of addiction symptoms (Csibi et al., 2018). Griffiths (2005) proposed salience, modification, mood, tolerance, withdrawal, conflict, and relapse as being the six of main criteria to assess problematic smartphone use in general (Chen et al., 2020). This is consistent with previous studies (e.g., Bányai et al., 2017; Csibi et al., 2018; Monacis et al., 2017; Sha et al., 2019) where problematic smartphone use was associated with (and can have an impact on) individual’s psychological distress (Chen et al., 2020).

The present study translated and validated the English version of the SABAS into the Bahasa Indonesian-version which was successfully adapted for use in the Indonesian cultural context. Based on the research findings, the Bahasa Indonesian version of the SABAS was shown to be a valid and reliable psychometric scale. The English version of the SABAS underwent a gradual translation process that involved Indonesian language and subject specialist experts. Therefore, the Bahasa Indonesian version of the SABAS scale can be disseminated and can be used to assess the risk of developing smartphone addiction. This is because SABAS has six items that assess an individual’s dependence on smartphone applications.

## 5 Limitations, conclusions, and directions for further research

There are some limitations to the present study. First, the participants were recruited using a convenience sampling method and the sample was arguably homogeneous in relation to the university students recruited. Therefore, the generalizability of the present study to all Indonesian university students could be considered sub-optimal. Future studies need to increase the diversity of the sample to provide additional psychometric information concerning the Indonesian SABAS. Second, the present study did not assess psychometric properties of the Indonesian SABAS as thoroughly as it could have. For example, there was no follow-up to assess test-retest reliability. Future studies are needed to examine additional psychometric properties of the Indonesian SABAS. Third, the SABAS is a self-report scale and it is possible that the participants might have provided biased responses due to social desirability. Additionally, the other scales used (i.e., DASS-21 and NMPQ) were also both self-report instruments. Therefore, single-rater biases may have occurred. Future studies may consider using other external measures to objectively assess smartphone use (e.g., time spent on smartphone use assessed via a smartphone app) (Kwok et al., 2022) to examine the concurrent validity of the SABAS.

The psychometric testing of the Indonesian SABAS found support for its linguistic validity and preliminary reliability. However, additional evidence using different psychometric methods is needed to strengthen the use of SABAS in Indonesia (e.g., testing known-group validity of the SABAS, evaluating the test-retest reliability of the SABAS, carrying out Rasch analysis to get more in-depth information about the scale at item level). Overall, the results of the present study indicate that the Indonesian version of SABAS is a valid and reliable instrument that can be used for assessing the risk of smartphone application-based addiction among college students in Indonesia.

## Declarations

### Author contribution statement

Ira Nurmala, PhD; Siti Rahayu Nadhiroh; Iqbal Pramukti: Conceived and designed the experiments; Performed the experiments; Analyzed and interpreted the data; Contributed reagents, materials, analysis tools or data; Wrote the paper.

Laila Wahyuning Tyas; Afina Puspita Zari: Performed the experiments; Analyzed and interpreted the data; Wrote the paper.

Mark D. Griffiths: Analyzed and interpreted the data; Wrote the paper.

Chung-Ying Lin: Conceived and designed the experiments; Analyzed and interpreted the data; Contributed reagents, materials, analysis tools or data; Wrote the paper.

### Funding statement

Dr Ira Nurmala was supported by Universitas Airlangga [1314/UN3.15/PT/2021].

### Data availability statement

Data are available on reasonable request to the corresponding author.

### Declaration of interest’s statement

The authors declare no conflict of interest.

### Additional information

No additional information is available for this paper.

## Appendix A Supplementary data

The Indonesia Version of SABAS_Ira Nurmala_Checklist The Indonesia Version of SABAS_Ira Nurmala_Checklist

SABAS Indonesian Version SABAS Indonesian Version

SABAS English Version SABAS English Version

SABAS 478 Respondent-Heliyon SABAS 478 Respondent-Heliyon

## References

[bib1] Arthy C.C., Effendy E., Amin M.M., Loebis B., Camellia V., Husada M.S. (2019). Indonesian version of addiction rating scale of smartphone usage adapted from smartphone addiction scale-short version (SAS-SV) in junior high school. Open Access Macedonian J. Med. Sci..

[bib2] Bányai F., Zsila Á., Király O., Maraz A., Elekes Z., Griffiths M.D., Andreassen C.S., Demetrovics Z. (2017). Problematic social media use: results from a large-scale nationally representative adolescent sample. PLoS One.

[bib3] Beaton D.E., Bombardier C., Guillemin F., Ferraz M.B. (2000). Guidelines for the process of cross-cultural adaptation of self-report measures. Spine.

[bib4] BPS-Statistics Indonesia (2019).

[bib5] BPS-Statistics Indonesia (2020).

[bib6] Brand M., Young K.S., Laier C., Wölfling K., Potenza M.N. (2016). Integrating psychological and neurobiological considerations regarding the development and maintenance of specific Internet-use disorders: an Interaction of Person-Affect-Cognition-Execution (I-PACE) model. Neurosci. Biobehav. Rev..

[bib7] Chang K.-C., Chang Y.-H., Yen C.-F., Chen J.-S., Chen P.-J., Lin C.-Y., Griffiths M.D., Potenza M.N., Pakpour A.H. (2022). A longitudinal study of the effects of problematic smartphone use on social functioning among people with schizophrenia: mediating roles for sleep quality and self-stigma. J. Behav. Addict..

[bib8] Chang Y.H., Chang K.C., Hou W.L., Lin C.Y., Griffiths M.D. (2020). Internet gaming as a coping method among schizophrenic patients facing psychological distress. J. Behav. Addict..

[bib9] Chen I.H., Ahorsu D.K., Pakpour A.H., Griffiths M.D., Lin C.Y., Chen C.Y. (2020). Psychometric properties of three simplified Chinese online-related addictive behavior instruments among mainland Chinese primary school students. Front. Psychiatr..

[bib10] Chen I.H., Chen C.Y., Liu C.H., Ahorsu D.K., Griffiths M.D., Chen Y.P., Kuo Y.J., Lin C.Y., Pakpour A.H., Wang S.M. (2021). Internet addiction and psychological distress among Chinese schoolchildren before and during the COVID-19 outbreak: a latent class analysis. J. Behav. Addict..

[bib11] Chen I.H., Chen C.Y., Pakpour A.H., Griffiths M.D., Lin C.Y., Li X.D., Tsang H.W.H. (2021). Problematic internet-related behaviors mediate the associations between levels of internet engagement and distress among schoolchildren during COVID-19 lockdown: a longitudinal structural equation modeling study. J. Behav. Addict..

[bib12] Chen I.H., Pakpour A.H., Leung H., Potenza M.N., Su J.A., Lin C.Y., Griffiths M.D. (2020). Comparing generalized and specific problematic smartphone/internet use: longitudinal relationships between smartphone applicationbased addiction and social media addiction and psychological distress. J. Behav. Addict..

[bib13] Chen I.H., Strong C., Lin Y.C., Tsai M.C., Leung H., Lin C.Y., Pakpour A.H., Griffiths M.D. (2020). Time invariance of three ultra-brief internet-related instruments: smartphone application-based addiction scale (SABAS), bergen social media addiction scale (BSMAS), and the nine-item internet gaming disorder scale- short form (IGDS-SF9) (study Part B). Addict. Behav..

[bib14] Cheung H., Mazerolle L., Possingham H.P., Tam K.P., Biggs D. (2020). A methodological guide for translating study instruments in cross-cultural research: adapting the ‘connectedness to nature’ scale into Chinese. Methods Ecol. Evol..

[bib15] Ching S.M., Yee A., Ramachandran V., Lim S.M.S., Sulaiman W.A.W., Foo Y.L., Hoo F.K. (2015). Validation of a Malay version of the smartphone addiction scale among medical students in Malaysia. PLoS One.

[bib16] Coker A.O., Coker O.O., Sanni D. (2018). Psychometric properties of the 21-item depression anxiety stress scale (DASS-21). African Res. Review.

[bib17] Csibi S., Demetrovics Z., Szabó A. (2016). Development and psychometric validation of the brief smartphone addiction scale (BSAS) with schoolchidren. Psychiatr. Hung..

[bib18] Csibi S., Griffiths M.D., Cook B., Demetrovics Z., Szabo A. (2018). The psychometric properties of the smartphone application-based addiction scale (SABAS). Int. J. Ment. Health Addiction.

[bib19] Elhai J.D., Levine J.C., Dvorak R.D., Hall B.J. (2017). Non-social features of smartphone use are most related to depression, anxiety and problematic smartphone use. Comput. Hum. Behav..

[bib20] Galhardo A., Loureiro D., Raimundo E., Massano-Cardoso I., Cunha M. (2020). Assessing nomophobia: validation study of the European Portuguese version of the nomophobia questionnaire. Community Ment. Health J..

[bib21] Griffiths M. (2005). A ‘components’ model of addiction within a biopsychosocial framework. J. Subst. Use.

[bib22] Hanafi E., Siste K., Wiguna T., Kusumadewi I., Nasrun M.W. (2019). Temperament profile and its association with the vulnerability to smartphone addiction of medical students in Indonesia. PLoS One.

[bib62] Herrero Juan, Torres Andrea, Vivas Pep, Urueña Alberto (2019). Smartphone Addiction and Social Support: A Three-year Longitudinal Study. Psychosocial Intervention.

[bib23] Hye J., Id P., Id M.P. (2021). Smartphone use patterns and problematic smartphone use among preschool children. PLoS One.

[bib24] Jayani D.H. (2021). https://databoks.katadata.co.id/datapublish/2021/05/24/proporsi-populasi-generasi-z-dan-milenial-terbesar-di-indonesia.

[bib25] Johnson O.A., Olaniyi S.F., John S., Sheila O., Daniel O., Imam A., Olaiya A.P. (2020). Baseline and postintervention assessment of sexual violence and condom use among female sex workers in a semiurban African community. Soc Health Behav..

[bib26] Kim S.-S., Reed P.G., Kang Y., Oh J. (2012). Translation and psychometric testing of the Korean versions of the spiritual perspective scale and the self-transcendence scale in Korean elders. J. Korean Academy Nursing.

[bib27] Kline R.B. (2011).

[bib28] Kwok C., Poon K.Y., Fung X.C.C. (2022). The effects of internet gaming and social media use on physical activity , sleep , quality of life , and academic performance among university students in Hong Kong: a preliminary study. Asian J Soc Health Behav..

[bib29] Kwon M., Lee J.Y., Won W.Y., Park J.W., Min J.A., Hahn C., Gu X., Choi J.H., Kim D.J. (2013). Development and validation of a smartphone addiction scale (SAS). PLoS One.

[bib30] Kwon Y.S., Paek K.S. (2016). The influence of smartphone addiction on depression and communication competence among college students. Indian J. Sci. Techn..

[bib52] Le M.T.H., Tran T.D., Holton S., Nguyen H.T., Wolfe R., Fisher J. (2020). Reliability, convergent validity and factor structure of the DASS-21 in a sample of Vietnamese adolescents. J. Affect. Disord..

[bib31] Lee S., Kim M., Mendoza J.S., McDonough I.M. (2018). Addicted to cellphones: exploring the psychometric properties between the nomophobia questionnaire and obsessiveness in college students. Heliyon.

[bib32] Leung H., Pakpour A.H., Strong C., Lin Y.C., Tsai M.C., Griffiths M.D., Lin C.Y., Chen I.H. (2020). Measurement invariance across young adults from Hong Kong and taiwan among three internet-related addiction scales: bergen social media addiction scale (BSMAS), smartphone application-based addiction scale (SABAS), and internet gaming disorder scale-short. Addict. Behav..

[bib33] Lin C.Y., Broström A., Griffiths M.D., Pakpour A.H. (2020). Psychometric evaluation of the Persian eHealth Literacy Scale (eHEALS) among elder Iranians with heart failure. Eval. Health Prof..

[bib34] Lin C.Y., Broström A., Nilsen P., Griffiths M.D., Pakpour A.H. (2017). Psychometric validation of the Persian bergen social media addiction scale using classic test theory and Rasch models. J. Behav. Addict..

[bib35] Lin C.Y., Griffiths M.D., Pakpour A.H. (2018). Psychometric evaluation of Persian Nomophobia Questionnaire: differential item functioning and measurement invariance across gender. J. Behav. Addict..

[bib36] Lin C.Y., Imani V., Broström A., Nilsen P., Fung X.C.C., Griffiths M.D., Pakpour A.H. (2019). Smartphone application-based addiction among Iranian adolescents: a psychometric study. Int. J. Ment. Health Addiction.

[bib37] Lopez-Fernandez O. (2017). Short version of the Smartphone Addiction Scale adapted to Spanish and French: towards a cross-cultural research in problematic mobile phone use. Addict. Behav..

[bib38] Ministry of Communication and Information (2019).

[bib39] Monacis L., De Palo V., Griffiths M.D., Sinatra M. (2017). Social networking addiction, attachment style, and validation of the Italian version of the Bergen Social Media Addiction Scale. J. Behav. Addict..

[bib40] Ndağ Y.A., Ünal A. (2019). Adaptation of application-based smartphone addiction scale to Turkish cultures. Sakarya Univ. J. Edu..

[bib41] Nejati B., Fan C.W., Boone W.J., Griffiths M.D., Lin C.Y., Pakpour A.H. (2021). Validating the Persian Intuitive Eating Scale-2 among breast cancer survivors who are overweight/obese. Eval. Health Prof..

[bib42] Nunnally J.C. (1978).

[bib43] Onie S., Kirana A.C., Alfian A., Mustika N.P., Adesla V., Ibrahim R. (2020).

[bib44] Poon L.Y.J., Tsang H.W.H., Chan T.Y.J., Man S.W.T., Ng L.Y., Wong Y.L.E., Lin C.Y., Chien C.W., Griffiths M.D., Pontes H.M., Pakpour A.H. (2021). Psychometric properties of the internet gaming disorder scale-short-form (IGDS9-SF): systematic review. J. Med. Internet Res..

[bib61] Purwaningsih Eni, Nurmala Ira (2021). The Impact of Online Game Addiction on Adolescent Mental Health: A Systematic Review and Meta-analysis. Open Access Macedonian Journal of Medical Sciences.

[bib45] Rangka I.B., Prasetyaningtyas W.E., Ifdil I., Ardi Z., Suranata K., Winingsih E., Sofyan A., Irawan M., Arjanto P., Muslifar R., Wijaya R.S. (2018). Measuring psychometric properties of the Indonesian version of the NoMoPhobia Questionnaire (NMPQ): insight from Rasch measurement tool. J. Phys. Conf..

[bib46] Rashvand H.F., Hsiao K.F. (2015). Smartphone intelligent applications: a brief review. Multimed. Syst..

[bib47] Rosseel Y. (2012). lavaan : an R package for structural equation modeling. J. Stat. Software.

[bib48] Sha P., Sariyska R., Riedl R., Lachmann B., Montag C. (2019). Linking internet communication and smartphone use disorder by taking a closer look at the Facebook and WhatsApp applications. Addict. Behav. Reports.

[bib49] Soraci P., Ferrari A., Antonino U., Griffiths M.D. (2021). Psychometric properties of the Italian version of the smartphone application-based addiction scale (SABAS). Int. J. Ment. Health Addiction.

[bib50] Stone R.C. (2020).

[bib51] Szabo A., Csibi S., Demetrovics Z. (2017). Development and validation of a smartphone deprivation scale (SDS) for use with schoolchildren. J. Behav. Addict..

[bib53] Vally Z., Alowais A. (2020). Assessing risk for smartphone addiction: validation of an Arabic version of the smartphone application-based addiction scale. Int. J. Ment. Health Addiction.

[bib54] Wang J.L., Wang H.Z., Gaskin J., Wang L.H. (2015). The role of stress and motivation in problematic smartphone use among college students. Comput. Hum. Behav..

[bib55] Wang K., Shi H.-S., Geng F.-L., Zou L.-Q., Tan S.-P., Wang Y., Neumann D.L., Shum D.H.K., Chan R.C.K. (2016). Cross-cultural validation of the depression anxiety stress scale–21 in China. Psychol. Assess..

[bib56] Wu T.Y., Lin C.Y., Årestedt K., Griffiths M.D., Broström A., Pakpour A.H. (2017). Psychometric validation of the Persian nine-item Internet Gaming Disorder Scale - short Form: does gender and hours spent online Gaming affect the interpretations of item descriptions?. J. Behav. Addict..

[bib57] Yam C.W., Pakpour A.H., Griffiths M.D., Yau W.Y., Lo C.L.M., Ng J.M.T., Lin C.Y., Leung H. (2019). Psychometric testing of three Chinese online-related addictive behavior instruments among Hong Kong university students. Psychiatr. Q..

[bib58] Yildirim C., Correia A.P. (2015). Exploring the dimensions of nomophobia: development and validation of a self-reported questionnaire. Comput. Hum. Behav..

[bib59] Yildirim C., Sumuer E., Adnan M., Yildirim S. (2016). A growing fear: prevalence of nomophobia among Turkish college students. Inf. Dev..

[bib60] Yuk H.D., Kim J.J., Ku J.H., Kwak C., Kim H.H., Jeong C.W. (2020). Korean version of the convalescence and recovery evaluation: translation and linguistic validation. Prostate International.

