# Supplementary material for: Reliability and validity study of the Indonesian Smartphone Application-Based Addiction Scale (SABAS) among college students
Source: Heliyon. 2022 Aug 24;8(8):e10403. doi: 10.1016/j.heliyon.2022.e10403 (PMC9449775; doi:10.1016/j.heliyon.2022.e10403)
Supplement: SABAS Indonesian Version [file mmc2.docx]

**Smartphone Application Based Addiction (SABAS)**

| **Items** | **Strongly disagree (1)** | **Disagree**  **(2)** | **Slightly agree (3)** | **Slightly agree (4)** | **Agree**  **(5)** | **Strongly agree (6)** |
| --- | --- | --- | --- | --- | --- | --- |
| 1. My smartphone is the most important thing in my life |  |  |  |  |  |  |
| 2. My smartphone use results in conflicts |  |  |  |  |  |  |
| 3. Preoccupying myself with my smartphone is a way of changing my mood |  |  |  |  |  |  |
| 4. I fiddle around more and more with my smartphone |  |  |  |  |  |  |
| 5. If I cannot use my smartphone when I feel like, I feel sad |  |  |  |  |  |  |
| 6. If I try to cut the time I use my smartphone, I end up using it as much or more than before |  |  |  |  |  |  |
