# Supplementary material for: Reliability and validity study of the Indonesian Smartphone Application-Based Addiction Scale (SABAS) among college students
Source: Heliyon. 2022 Aug 24;8(8):e10403. doi: 10.1016/j.heliyon.2022.e10403 (PMC9449775; doi:10.1016/j.heliyon.2022.e10403)
Supplement: SABAS English Version [file mmc3.docx]

**Smartphone Application Based Addiction (SABAS) – Indonesian version**

| **Pertanyaan** | **Sangat Tidak Setuju (1)** | **Tidak Setuju (2)** | **Sedikit Tidak Setuju (3)** | **Sedikit Setuju (4)** | **Setuju**  **(5)** | **Sangat Setuju (6)** |
| --- | --- | --- | --- | --- | --- | --- |
| 1. *Smartphone* adalah hal terpenting dalam hidup saya |  |  |  |  |  |  |
| 1. Aktivitas saya menggunakan *smartphone* menyebabkan konflik |  |  |  |  |  |  |
| 1. Menyibukkan diri dengan *smartphone* adalah cara untuk mengubah suasana hati saya |  |  |  |  |  |  |
| 1. Saya menghabiskan waktu terus-menerus hanya untuk bermain *smartphone* |  |  |  |  |  |  |
| 1. Ketika saya tidak dapat menggunakan *smartphone* saat ingin, saya merasa sedih |  |  |  |  |  |  |
| 1. Jika saya mencoba mengurangi waktu menggunakan *smartphone*, saya semakin menggunakannya lebih lama atau lebih sering dari sebelumnya |  |  |  |  |  |  |
