# Supplementary material for: Reliability and validity study of the Indonesian Smartphone Application-Based Addiction Scale (SABAS) among college students
Source: Heliyon. 2022 Aug 24;8(8):e10403. doi: 10.1016/j.heliyon.2022.e10403 (PMC9449775; doi:10.1016/j.heliyon.2022.e10403)
Supplement: SABAS 478 Respondent-Heliyon [file mmc4.doc]

***Heliyon***

**Manuscript Checklist**

Submission checklist The following list will be useful during the final checking of an article prior to sending it to the journal for review. Please consult this Guide for Authors for further details of any item. Ensure that the following items are present:

| All manuscript and supplementary material files have been uploaded | ✓ |
| --- | --- |
| Author names and their affiliations have been provided. | ✓ |
| One author has been designated as corresponding author | ✓ |
| The manuscript title is short and informative. | ✓ |
| The abstract can be read as stand-alone text and does not exceed 300 words. | ✓ |
| The manuscript file contains all essential sections (the order can vary): Title, Abstract, Introduction, Materials and Methods, Results, Discussion, References, Figure Captions, Tables. | ✓ |
| Manuscript has been checked for spelling and grammar. | ✓ |
| All references mentioned in the References list are cited in the text, and vice versa. | ✓ |
| Artwork source files have been provided at the appropriate size and resolution. | ✓ |
| Relevant subject areas have been selected and keywords describing the article have been provided. | ✓ |
| All necessary declarations have been made. | ✓ |
